# GLIM in diagnosing malnutrition and predicting outcome in ambulatory patients with head and neck cancer

**DOI:** 10.3389/fnut.2022.1030619

**Published:** 2022-11-22

**Authors:** Helena Kristiina Orell, Anne Katariina Pohju, Pia Osterlund, Ursula Sonja Schwab, Paula Ravasco, Antti Mäkitie

**Affiliations:** ^1^Clinical Nutrition Unit, Internal Medicine and Rehabilitation, Helsinki University Hospital, University of Helsinki, Helsinki, Finland; ^2^Institute of Clinical Medicine, School of Medicine, Faculty of Health Sciences, University of Eastern Finland, Kuopio, Finland; ^3^Department of Oncology, Tampere University Hospital, Tampere, Finland; ^4^Department of Oncology/GI-cancer, Karolinska University Hospital, Stockholm, Finland; ^5^Department of Oncology/Pathology, Karolinska Institutet, Stockholm, Finland; ^6^Department of Oncology, Helsinki University Hospital, Helsinki, Finland; ^7^Department of Medicine, Endocrinology and Clinical Nutrition, Kuopio University Hospital, Kuopio, Sweden; ^8^Universidade Católica Portuguesa, Católica Medical School and Centre for Interdisciplinary Research in Health (CIIS), Lisbon, Portugal; ^9^Clinical Research Unit, Egas Moniz Interdisciplinary Research Center, Almada, Portugal; ^10^Research Program in Systems Oncology, Faculty of Medicine, University of Helsinki, Helsinki, Finland; ^11^Division of Ear, Nose and Throat Diseases, Department of Clinical Sciences, Intervention and Technology, Karolinska University Hospital, Stockholm, Sweden; ^12^Department of Otorhinolaryngology-Head and Neck Surgery, Helsinki University Hospital, University of Helsinki, Helsinki, Finland

**Keywords:** nutrition status, nutrition status assessment, nutritional risk, survival, malnutrition, nutritional risk screening 2002, Patient-Generated Subjective Global Assessment, head and neck cancer

## Abstract

**Aim:**

This study aimed to determine the prevalence of malnutrition in a head and neck cancer (HNC) population according to the Global Leadership Initiative on Malnutrition (GLIM) criteria and to assess its relation to survival. The secondary aim was to compare GLIM criteria to Patient–Generated Subjective Global Assessment (PG–SGA) and Nutritional Risk Screening 2002 (NRS 2002) methods.

**Methods:**

The assessment was performed in a series of 65 curative patients with newly diagnosed HNC in a nutrition intervention study. Malnutrition was defined as PG-SGA classes BC and nutritional risk as NRS 2002 score ≥3 and was retrospectively diagnosed with GLIM criteria in prospectively collected data at diagnosis. Sensitivity, specificity, and kappa (κ) were analyzed. Predictive accuracy was assessed by calculating the area under curve (AUC) b y receiver operating characteristic (ROC) analysis. Kaplan–Meier and Cox regression analyses were used to evaluate association between malnutrition and overall survival (OS), and disease-free survival (DFS).

**Results:**

GLIM-defined malnutrition was present in 37% (24/65) of patients. The GLIM showed 77% sensitivity and 84% specificity with agreement of κ = 0.60 and accuracy of AUC = 0.80 (*p* < 0.001) with PG-SGA and slightly higher sensitivity (83%) with NRS 2002 (κ = 0.58). Patients with GLIM-defined malnutrition had shorter OS (56 vs. 72 months, HR 2.26, 95% CI 1.07–4.77, *p* = 0.034) and DFS (37 vs. 66 months, HR 2.01, 95% CI 0.99–4.09, *p* = 0.054), than well-nourished patients. The adjusted HR was 2.53 (95% CI 1.14–5.47, *p* = 0.023) for OS and 2.10 (95% CI 0.98–4.48, *p* = 0.056) for DFS in patients with GLIM-defined malnutrition.

**Conclusion:**

A substantial proportion of HNC patients were diagnosed with malnutrition according to the GLIM criteria and this showed a moderate agreement with NRS 2002- and PG–SGA-defined malnutrition. Even though the GLIM criteria had strong association with OS, its diagnostic value was poor. Therefore, the GLIM criteria seem potential for malnutrition diagnostics and outcome prediction in the HNC patient population. Furthermore, NRS 2002 score ≥3 indicates high nutritional risk in this patient group.

## Introduction

Malnutrition is defined as an acute or chronic state of impaired nutritional status, in which a combination of varying degrees of nutrition intake and inflammatory activity have led to harmful changes in body composition and function (1). Prevention, early identification of patients at risk, accurate diagnosis, personalized nutrition interventions, and follow–up are cornerstones of the management of malnutrition and the prevention of its unfavorable effects on treatment complications, patients' quality of life, and survival (2–6). However, variation in nutritional status criterion makes the comparison of the effectiveness of nutrition interventions across different studies challenging. Consequently, the Global Leadership Initiative on Malnutrition (GLIM) working group published in 2018 a global consensus recommendation on the criteria to be used for the identification of protein–energy malnutrition in adults (7). Since then several studies have validated these criteria in various patient cohorts, including head and neck cancer (HNC) (8–11).The GLIM criteria have often been compared either with Subjective Global Assessment or Patient-Generated Subjective Global Assessment (SGA or PG–SGA), which are judged to be the most validated standardized assessment tools of malnutrition (12). So far the GLIM criteria have shown to be an accurate, sensitive, and specific malnutrition diagnostic tool in ambulatory cancer care and in-patient settings. Furthermore, the GLIM criteria have shown high inter–rater reliability in patients with HNC (8). However, the GLIM criteria have shown only a fair agreement with the SGA (10, 11).

In 2020, almost 880,000 new cases of HNC (e.g., lip and oral cavity, larynx, nasopharynx, oropharynx, and hypopharynx) and 445,000 associated deaths were observed worldwide with an overall 5-year survival rate of around 50% (13). Throughout the HNC journey, around 11–85% of patients present with malnutrition when assessed either with PG–SGA or with the GLIM criteria (8–10, 14, 15). Nutrition care plays a crucial role for patients with HNC since tumor itself and cancer treatments cause substantial eating and swallowing difficulties resulting in decreased food intake and deteriorated nutritional status which can be effectively managed by nutritional interventions (15, 16). This warrants further attention as malnutrition reduces treatment efficacy? (2, 4), quality of life (2), and survival (3, 4), as well as increases complications (4). Moreover, a GLIM-defined malnutrition diagnosis associates with lower BMI (8) and impaired quality of life (17) and PG-SGA-defined malnutrition with shorter overall survival (OS) and disease–free survival (DFS), as we have previously shown in patients with HNC (15).

The GLIM criteria have eight possible combinations to classify patients as malnourished, and controversies in sensitivity and specificity between these combinations exist (8, 9, 12). As the GLIM criteria are based on consensus, further evidence is required for validation and reliability of testing in a variety of healthcare sectors and populations with diverse persons using these criteria (18). So far only two studies have used the GLIM criteria to diagnose malnutrition in HNC, and they have shown a prevalence of malnutrition in 11–32% of patients (8, 9). The prevalence of GLIM-defined malnutrition has been 24–70% in patients with other cancers (19–23). Furthermore, the GLIM criteria have shown their predictive value with respect to survival in various clinical conditions (10, 19–22, 24).

Nutritional risk screening 2002 (NRS 2002) is a method to obtain patients who have a risk to develop protein-energy malnutrition (25). Usually, the score ≥3 indicates nutritional risk and a need for further assessment of nutritional status either with PG-SGA or GLIM. However, in a nutritionally more vulnerable patient group such as HNC, NRS 2002 score ≥3 may already indicate malnutrition as we showed in our previous study (14).

This study aimed to determine (1) the prevalence of malnutrition according to the GLIM criteria at diagnosis of HNC; (2) the reliability of using the GLIM criteria to identify malnutrition compared to the current reference standard, namely, the PG–SGA and to the NRS 2002; and (3) the associations between the GLIM criteria and survival, and the predictive validity of the GLIM criteria with respect to survival.

## Materials and methods

This is a retrospective analysis of baseline measurements collected during a previously published randomized controlled study of adult HNC patients (14) at the Department of Otorhinolaryngology, Head and Neck Surgery, Helsinki University Hospital (HUS), Finland. Ambulatory, 18–80-year-old patients with a primary locally advanced squamous cell carcinoma of the oral cavity, oropharynx, hypopharynx, nasopharynx, or larynx were eligible for inclusion. A total of 65 patients with HNC were included. All patients were under nutritional surveillance and were offered nutritional treatment when indicated (15).

Clinical prospectively collected data included age, gender, tumor histopathology, site and stage classification, and cancer treatment (definitive chemoradiotherapy, definitive radiotherapy, surgery, surgery with radiotherapy, or surgery with chemoradiotherapy). All nutritional measurements and subjective assessments, except GLIM, were performed prospectively at the time of diagnosis before surgery or adjuvant cancer treatment.

Body mass index (BMI) was calculated from the patient-reported height and measured body weight and was further categorized according to age as underweight (<18.5 kg/m^2^ if <65 years or <22 kg/m^2^ if ≥65 years); healthy weight (18.5–24.9 kg/m^2^ if <65 years or 22–27 kg/m^2^ if ≥65 years) or overweight (≥25 kg/m^2^ if <65 years or >27 kg/m^2^ if ≥65 years). Body composition was analyzed with bioimpedance (BIA) using a single frequency (50 kHz) two–terminal bio–impedance meter (Bodystat Ltd^®^, Isle of Man, UK) performed according to a standard procedure.

Nutritional status was assessed using the PG–SGA (26, 27) with classes B and C indicating malnutrition. Nutritional risk was current study the NRS 2002 score ≥3 was set to indicate nutritional risk (25). Patient-Generated SGA was considered as the reference method to identify protein-energy malnutrition, as this is what GLIM was designed to identify (12). Permission for the full form of scored PG-SGA© was received from Pt-Global (http://pt-global.org/). The English PG-SGA version 2001 was translated into Finnish through backward translation by a medical doctor (PÖ) and dietitian (HO) in our research group. PÖ translated it to Finish and then HO translated it back to English whereupon they both translated it to Finnish. No methodical discrepancies were observed. Research supervisors (PÖ and AM) accepted the final translation. PÖ supervised the subjective assessment of body composition, and the execution of PG-SGA. The research dietitian (HO) performed both the patient and the professional components of PG-SGA for all patients.

A GLIM diagnosis of malnutrition was assigned retrospectively when one phenotypic and one etiologic criterion were present and categorized as “malnourished” or “not malnourished” with minimum of one criteria of each existing (7). As a phenotypic criterion for malnutrition, we used body weight loss (>5% within the past 6 months), BMI (<20 kg/m^2^ if age <70 years or <22 kg/m^2^ if ≥70 years), and fat–free mass index (FFMI) by BIA (<17 kg/m^2^ for men and <15 kg/m^2^ for women) was used as an operationalization of the criterium “reduced muscle mass”. The etiologic criterion was either reduced food intake defined as ≤ 50% of estimated need or CRP >5 mg/L as a proxy for inflammation (8, 9). Food intake was compared to patients' usual eating and intake was categorized subjectively as ≤ 50% of estimated need if the patient had tumor-induced eating problems and therefore ate 50% less than normally or was unable to eat *per os*. Since all patients had a chronic active disease as per GLIM etiologic criterion, CRP was used as a more specific measure to define inflammation in line with previous studies (8, 9, 11). Cancer diagnosis itself is not recommended to be used for this etiologic criterion as it does not indicate the severity of the disease burden (12).

Patient outcome measures were collected at a median of 76 (IQR 71–81) months after the initial study date assessed by Kaplan-Meier and data were obtained from the electronic medical records. Data cut-off date was assigned as March 18th, 2015. Follow-up time and overall survival (OS) was calculated from the date of randomization (i.e., at diagnosis) to the date of the last visit or death by any cause. Disease–free survival (DFS) was calculated from the completion of treatment to the detection of cancer recurrence or death of any cause. There were no cancer events during cancer treatment. One patient with a second primary of esophagus cancer was excluded from the DFS analyses.

The research clinical dietitian (HO) performed nutritional status assessments, BIA measurements, and GLIM diagnostics for all patients. Permission for the full form of scored PG–SGA© was received from Pt–Global (https://pt-global.org/). The Finnish translation of NRS 2002 has shown substantial agreement (*k* = 0.8) with PG–SGA (14). The study design was approved by the Research Ethics Board at our institution and has a research permission (HUS/186/2021) granted by our institution. All patients gave a written informed consent.

### Statistical analyses

Descriptive statistics for all continuous variables were reported as median with inter–quartile range (IQR). Categorical variables were reported as frequencies and percentages. Construct (discriminant) validity was assessed using Chi–square test for categorical variables and Mann–Whitney's *U*-test for continuous variables.

Sensitivity, specificity, and positive (PPV) and negative predictive values (NPV) for the GLIM criteria against PG–SGA, NRS 2002, and survival were calculated from a contingency table. Rating of validity test statistics followed recommended cut points for sensitivity and specificity: the professional standard 80% for sensitivity and 60% for specificity were determined.

Assessment of agreement between the GLIM criteria, the PG–SGA and survival used the Kappa statistics (*κ*). Values 0.81–0.99 represented “excellent” agreement, 0.61–0.80 “substantial”, 0.41–0.60 “moderate” and <0.41 “poor to fair” agreement. The professional standard for kappa was set to >0.60.

Predictive accuracy between the GLIM criteria, PG-SGA and survival was assessed by calculating the area under curve (AUC) by receiver operating characteristic (ROC) analysis. Accuracy was considered very good if the ROC AUC was >0.9, good if 0.8–0.9, fair if 0.7–0.8, poor if 0.6–0.7 and not better than chance if <0.6.

Overall survival and DFS were calculated using the Kaplan–Meier method and the log-rank test. Cox proportional univariable hazards analyses (hazard ratio, HR) were performed to determine the association between GLIM-defined malnutrition diagnosis and mortality. Multivariable analyses were adjusted for age ( ≤ 65 vs. >65 years), gender, stage (I–III vs. IV), GLIM-defined malnutrition (not malnourished vs. malnourished), and smoking (<10 vs. ≥10 pack years) based on available literature (28).

All statistical analyses were performed with SPSS, Version 27.0 (IBM corp., Armonk, NY, US). We set the statistical significance level to 5%.

## Results

The median age was 61 years (range 33–77) with 25% being 65 or older, and the male–to–female ratio was 3.3:1 (55 males, 15 females). Most patients had stage IV disease (*n* = 44, 68%) and were planned to receive either definitive chemoradiotherapy (65%), or either surgery alone or as a combination treatment (26%). Only 7.3% of patients had definitive radiotherapy. The median (IQR) follow-up time was 76 (71–81) months assessed by Kaplan-Meier. The descriptive data according to GLIM-defined malnutrition diagnosis are shown in Table 1.

**Table 1:** Descriptive data of 65 HNC patients stratified according to GLIM-defined malnutrition diagnosis.

	**GLIM-defined malnutrition diagnosis (*****n*** = **65)**		
	**Not malnourished, 41 (63.1%)**	**Malnourished, 24 (36.9%)**	***p*** **-value**
Age, years, median (IQR)	61 (55–64)	59.5 (57–64)	0.749
Men, *n* (%)	33 (80.5)	17 (70.8)	NS
**Nutritional parameters, median (IQR)**
Weight, kg	79.7 (67.0–90.1)	64.9 (56.2–77.4)	0.004
BMI, kg/m^2^	25.7 (23.0–28.0)	21.6 (20.1–23.8)	<0.001
FFMI, kg/m^2^	17.8 (16.1–19.3)	15.5 (14.2–16.2)	<0.001
Weight loss, kg	0.2 (1.2–0.9)	6.0 (4.4–8.4)	<0.001
Weight loss, %	0.2 (1.3–1.2)	9.3 (6.2–11.3)	<0.001
C–reactive protein, g/L	3.0 (3.0–9.0)	20.5 (9.0–53.5)	<0.001
Albumin, g/L	40.3 (38.0–42.2)	35.4 (31.6–40.0)	<0.001
Prealbumin, mg/L	275 (225–304)	180 (127–229)	<0.001
Hemoglobin, mg/L	142 (131–150)	134 (124–140)	0.015
**Weight status**, ***n*** **(%)**^**#**^
Underweight	4 (9.8)	6 (25.0)	NS
Healthy weight	17 (41.5)	15 (62.5)	NS
Overweight	20 (48.8)	3 (12.5)	0.003
**SG–PGA**, ***n*** **(%)**
Well–nourished (class A)	36 (87.8)	7 (29.2)	<0.001
Malnourished (class B or C)	5 (12.2)	17 (70.8)	<0.001
**NRS 2002**, ***n*** **(%)**
Not nutritionally at risk (score <3)	38 (92.7)	9 (37.5)	<0.001
Nutritionally at risk (score ≥3)	3 (7.3)	15 (62.5)	<0.001
**Smoking, pack years**
<10	25 (61.0)	17 (70.8)	NS
≥10	16 (39.0)	7 (29.2)	NS
**Survival, months, median (95% CI)**
OS	67 (58–77)	54 (15–93)	0.029^*^
DFS	60 (50–71)	21 (0–70)	0.047^*^
**OS status**, ***n*** **(%)**
Diseased	14 (34.1)	14 (58.3)	NS
Survivor	27 (65.9)	10 (41.7)	NS
**DFS status**, ***n*** **(%)**
Event	16 (39.0)	15 (62.5)	NS
Survivor	25 (61.0)	9 (37.5)	NS
**Tumor location**, ***n*** **(%)**
Oral cavity	6 (14.6)	6 (25.0)	NS
Oropharynx	16 (39.0)	7 (29.2)	NS
Hypopharynx	5 (12.2)	6 (25.0)	NS
Larynx	9 (22.0)	2 (8.3)	NS
Nasopharynx	5 (12.2)	2 (8.3)	NS
Unknown primary	0 (0)	1 (4.2)	NS
**Stage**, ***n*** **(%)**
I	5 (12.2)	0 (0)	NS
II	4 (9.8)	2 (8.3)	NS
III	10 (24.4)	2 (8.3)	NS
IV	22 (53.7)	20 (83.3)	0.016
**Planned mode of cancer treatment**, ***n*** **(%)**
Surgery alone or in combination	14 (34.1)	3 (12.5)	NS
Definitive radiotherapy	3 (7.3)	3 (12.5)	NS
Definitive chemoradiotherapy	24 (58.5)	18 (75.0)	NS

BMI, body mass index; DFS, disease–free survival; FFMI, fat–free mass index; GLIM, the Global Leadership Initiative on Malnutrition; IQR, interquartile range; NRS, nutritional risk screening; OS, overall survival; PG–SGA, Patient–generated Subjective Global Assessment, A = well-nourished, B= moderately and C = severely malnourished.

# Underweight, BMI <18.5 kg/m^2^ if <65 years or <22 kg/m^2^ if ≥65 years; healthy weight, BMI 18.5–24.9 kg/m^2^ if <65 years or 22–27 kg/m^2^ if ≥65 years; overweight, BMI ≥25 kg/m^2^ if <65 years or >27 kg/m^2^ if ≥65 years.

* Analyzed by Kaplan-Meier.

Of the 65 patients, 37% were malnourished according to the GLIM criteria and 34% according to PG–SGA, while nutritional risk according to NRS 2002 was seen in 28% of patients at the time of cancer diagnosis and before any cancer treatment. All nutritional parameters were statistically significantly lower in patients with GLIM-defined malnutrition than in those not malnourished. Table 2 shows the numbers of patients with each phenotypic and etiologic criterion of GLIM-defined malnutrition. The criterion of unintentional weight loss was met by 40%, low BMI by 18%, low FFMI by 52%, low food intake by 25%, and inflammation by 51% of patients. We found no statistically significant differences between phenotypic or etiologic criteria between deceased patients and survivors (data not shown).

**Table 2:** The prevalence of the phenotypic and etiologic GLIM criteria according to malnutrition diagnosis.

	**GLIM-defined malnutrition diagnosis (*****n*** = **65)**, ***n*** **(%)**		
**GLIM criteria**	**Not malnourished, 41 (63%)**	**Malnourished, 24 (37%)**	***p*** **-value**
**Phenotypic criteria**
Weight loss >5%	5 (12)	21 (88)	<0.001
Low BMI^*^	5 (12)	7 (29)	<0.001
Low FFMI^#^	13 (32)	21 (88)	<0.001
**Etiologic criteria**
Low food intake	1 (2)	15 (62)	<0.001
Presence of inflammation	10 (24)	23 (96)	<0.001

BMI body mass index; FFMI, fat–free mass index; GLIM, Global Leadership Initiative on Malnutrition.

* BMI <20 kg/m^2^ if age <70 years or <22 kg/m^2^ if ≥70 years.

# FFMI <17 kg/m^2^ for men and <15 kg/m^2^ for women was used as an operationalization of the criterium “reduced muscle mass”.

*p*-value by Fisher's Exact Test.

### Diagnostic value of the GLIM criteria

**Figure 1:**
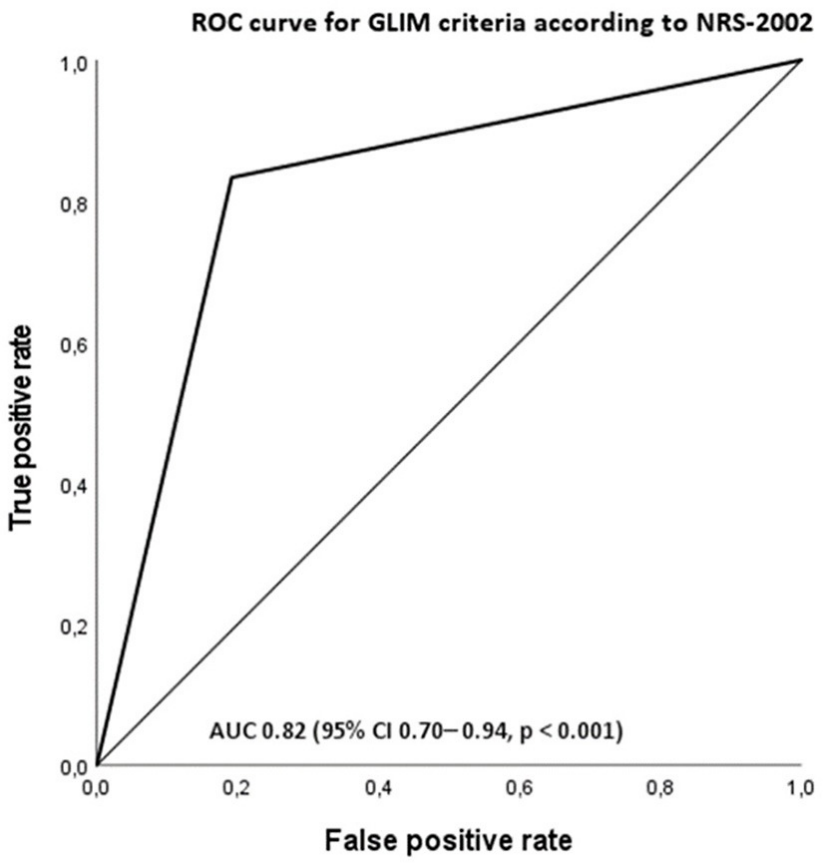
Receiver operating characteristic curve (ROC) for GLIM-diagnosed malnutrition as a measure of malnutrition according to patient-subjective global assessment (PG-SGA) group BC. The area under the curve (AUC) is 0.805 (95% CI 0.68–0.93, *p* < 0.001).

The agreement between GLIM and PG-SGA is shown in Table 3. When considering the PG-SGA as the reference method, the sensitivity of GLIM did not reach acceptable level (>0.80) while the specificity did (>0.60). The agreement between the PG-SGA and GLIM criteria was moderate according to the Kappa statistics (0.60) and the predictive value was fair according to AUC (Figure 1). The negative predictive value (NPV) was acceptable, but the positive predictive value was less than the acceptable level (>0.80).

**Table 3:** Diagnostic value of GLIM criteria in predicting malnutrition and survival.

**Reference method**	**GLIM criteria**						
	**Sensitivity (95% CI)**	**Specificity (95% CI)**	**PPV**	**NPV**	***k***	**AUC (95% CI)**	***p*** **-value**
PG–SGA BC	77.3 (57.1–90.8)	83.7 (70.7–92.4)	70.8	87.8	0.597	0.80 (0.68–0.93)	<0.001
NRS 2002 ≥3	83.3 (61.9–95.1)	80.9 (68.0–90.1)	62.5	92.7	0.582		
5-year OS	50.0 (32.2–67.8)	73.0 (57.3–85.2)	58.3	65.9	0.233	0.59 (0.44–0.74)	0.229
5-year DFS	48.4 (31.6–65.6)	73.5 (57.2–86.0)	62.5	61.0	0.221	0.62 (0.48–0.76)	0.116

AUC, area under curve; DFS, disease-free survival; GLIM, the Global Leadership Initiative on Malnutrition; *k*, Kappa correlation coefficient; NPV, negative predictive value; NRS, nutritional risk screening, ≥ 3 = nutritional risk; OS, overall survival; PG–SGA BC, Patient–generated Subjective Global Assessment; BC, moderately or severely malnourished; PPV, positive predictive value. *p*-value is for AUC.

### Association of the GLIM criteria and NRS 2002

The agreement between GLIM and NRS 2002 is shown in Table 3. When considering the NRS 2002 score ≥3 as the reference method, the sensitivity and specificity of GLIM did reach acceptable level. The agreement between the NRS 2002 and GLIM criteria was moderate according to the Kappa statistics (0.60). The negative predictive value (NPV) was acceptable, but the positive predictive value did not reach the acceptable level.

### Overall and disease-free survival

**Figure 2:**
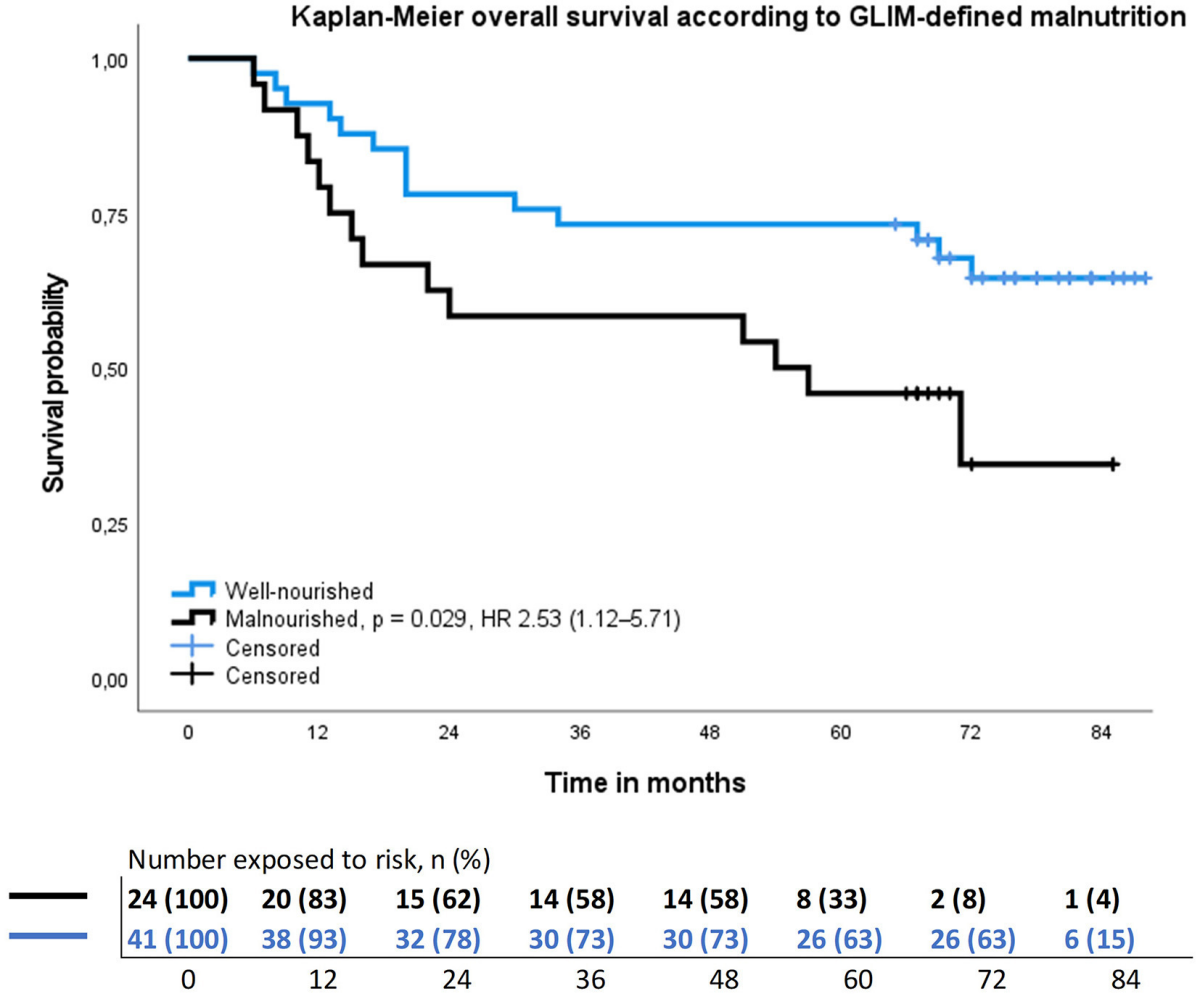
Results of the Kaplan–Meier overall survival analysis and hazard ratio for head and neck cancer patients stratified by GLIM–defined nutrition status. Hazard ratio adjusted to age, gender, stage, and smoking.

**Figure 3:**
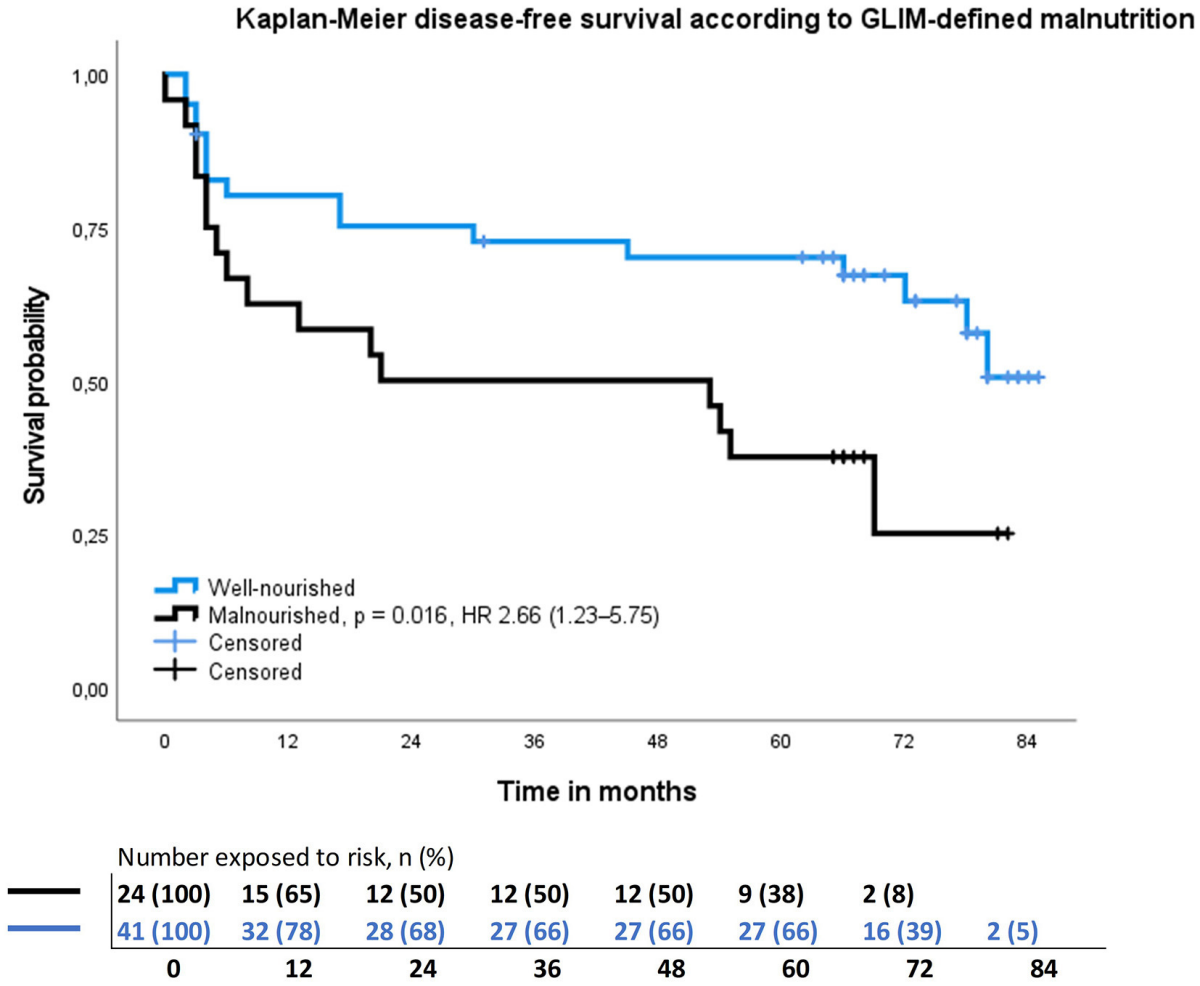
Results of the Kaplan–Meier disease-free survival analysis and hazard ratio for head and neck cancer patients stratified by GLIM–defined malnutrition. Hazard ratio adjusted to age, gender, stage, and smoking.

The 5-year OS rate was 57% (37/65) and DFS 52% (34/65) for all patients. Altogether 28 (43%) patients died during follow-up, of which 17 (26%) patients due to HNC, six due to other cancer, and five due to other causes. Malnourished patients had significantly lower OS (*p* = 0.029) and DFS (*p* = 0.047), than not malnourished patients (Table 1), as analyzed by Kaplan-Meier analysis (Figures 2, 3). Hazard ratios for OS and DFS according to Cox regression analysis are shown in Tables 4, 5. The association of malnutrition with OS and DFS was maintained when age, gender, stage, and smoking were added as covariates in adjusted multivariate models (Tables 4, 5). The accuracy of the GLIM criteria to predict OS and DFS was poor according to *k*-value and not better than chance according to AUC (Table 3).

**Table 4:** Univariable and multivariable regression analysis of overall survival in 65 HNC patients.

	**Univariable analysis**			**Multivariable analysis**		
**Variables**	**Hazard ratio**	**95% CI**	***p*** **-value**	**Hazard ratio**	**95% CI**	***p*** **-value**
**Age, years**
≤65	Reference					
>65	0.99	0.95–1.2	0.491	0.34	0.11–1.03	0.056
**Gender**
Female	Reference				
Male	2.15	0.74–6.2	0.157	2.36	0.77–7.20	0.133
**Stage**
I–III	Reference				
IV	0.88	0.41–1.91	0.747	0.51	0.22–1.18	0.115
**GLIM**
Not malnourished	Reference				
Malnourished	2.26	1.07–4.77	0.034	2.53	1.12–5.71	0.025
**Smoking, pack year**
<10	Reference				
≥10	4.26	1.48–12.32	0.007	3.32	1.11–9.91	0.031

CI, confidence interval; GLIM, Global Leadership Initiative on Malnutrition.

**Table 5:** Univariable and multivariable regression analysis of disease-free survival in 64^#^ HNC patients.

	**Univariable analysis**			**Multivariable analysis**		
**Variables**	**Hazard ratio**	**95% CI**	***p*** **-value**	**Hazard ratio**	**95% CI**	***p*** **-value**
**Age, years**
≤65	Reference				
> 65	0.52	0.2–1.36	0.185	0.49	0.18–1.36	0.170
**Gender**
Female	Reference				
Male	1.76	0.68–4.59	0.246	1.73	0.63–4.75	0.288
**Stage**
I–III	Reference				
IV	0.83	0.40–1.70	0.605	0.57	0.25–1.28	0.171
**GLIM**
Not malnourished	Reference				
Malnourished	2.01	0.99–4.09	0.054	2.10	0.98–4.48	0.056
**Smoking, pack years**
<10	Reference				
≥10	5.18	1.80–14.87	0.002	4.21	1.43–12.37	0.009

CI, confidence interval; GLIM Global Leadership Initiative on Malnutrition.

^#^One patient with second primary was excluded from the DFS analyses.

## Discussion

Our findings showed that the GLIM criteria form an accurate, sensitive, and specific malnutrition diagnostic method for the HNC population. However, the GLIM criteria showed poor diagnostic value in predicting 5-yr survival in this patient population. NRS 2002 score ≥3 showed to be an accurate tool to identify malnourished patients compared against the GLIM criteria, a finding supporting our previous study (14).

### Prevalence of malnutrition according to the GLIM criteria

Two previous studies have validated the GLIM criteria in patients with HNC. Prior to any cancer treatment, the prevalence of GLIM-defined malnutrition has been reported to vary from 11 to 23% and to increase up to 32% at the seventh week of HNC treatment (8, 9). Several factors might explain why our study showed a higher prevalence of GLIM-defined malnutrition than these two recent cross-sectional cohorts (8, 9). First, we used CRP as an objective measure for inflammation instead of the presence of metastatic disease, the latter of which may have resulted in some under–reporting of malnutrition in the Steer study (8). Second, Steer and colleagues assessed muscle–mass subjectively as opposed to our objective and more precise BIA analysis. Third, in the Einarsson et al. (9) study, patients were somewhat older, and Stage IV was seen in fewer patients (55%) compared with our study (65%). This high prevalence of stage IV disease indicates a more severe disease and consequently, a higher likelihood of dysphagia, cachexia, and thus higher prevalence of malnutrition already prior diagnosis (6, 29). Indeed, we have shown previously that a substantially high proportion of our patients had cachexia prior to diagnosis (3).

### Diagnostic value of the GLIM criteria

Since the publishing of the GLIM criteria, several validation studies have been conducted among medical, surgical, intensive care unit (ICU), and cancer patients (8–11, 30–35). Four studies report criterion validity from fair to good when GLIM criteria were compared with SGA. The agreement with kappa statistics has varied from 0.32 to 0.55 (10, 31, 35) in patients with cancer. A higher agreement (*k* = 0.85) has been seen among ICU patients (34). Sensitivity has varied from 61 to 92% and specificity from 73 to 93% in various patient cohorts (10, 11, 30, 32–34). Our results show moderate agreement, sensitivity and specificity, which are well in line with those studies conducted in cancer patients (10, 31, 35). Nevertheless, further prospective validation studies are needed to add knowledge on how to assess muscle mass and disease burden (i.e., inflammation) because the predictive validity of the GLIM criteria varies greatly (sensitivity 61–100%, specificity 55–98%) depending on the used criteria as shown in patients with surgery for gastrointestinal diseases (30).

The more precise criteria used to diagnose malnutrition in the current study may explain the better validity seen in our study compared with studies by Steer et al. (8), De Groot et al. (10), and Allard et al. (11). In addition, in the current study a clinical dietitian conducted nutrition assessment and GLIM diagnostics instead of trained coordinators or other staff (10, 11). The GLIM criteria have shown an excellent level of inter-rater agreement between two dietitians, a result suggesting that qualified medical personnel should perform GLIM diagnostics (8). Furthermore, different combinations of GLIM criteria have been compared and the best combinations seem to be either weight loss and high CRP or weight loss and low food intake, both of which we used in the present study (9, 11). A lack of consensus regarding how to accurately and practically measure and define reduced muscle mass and inflammatory burden caused by different diseases still exists, warranting further studies.

### Association of the GLIM criteria and NRS 2002

To the best of our knowledge, a comparison between NRS 2002 and the GLIM criteria has not been conducted in this specific patient population. Among hospitalized patients sensitivity (84%) and specificity (94%) were good between the GLIM criteria and NRS 2002 and the concordance in diagnosing malnutrition was substantial (κ = 0.784) (24). Even better results were obtained in 637 hospitalized cancer patients evaluated at admission; sensitivity 82%, specificity 98%, *κ* = 0.823 (36). In the current study GLIM showed high sensitivity and specificity with the NRS-2002 indicating that patients with NRS 2002 score ≥3 are at high nutritional risk and even malnourished as proposed in our previous study (14).

### Survival

The association of the GLIM criteria with survival has not been previously studied in this patient population but it has been shown that GLIM-defined malnutrition is an independent prognostic factor of survival in cancer patients in general (10, 37), and in patients with gastrointestinal cancer (20), hematologic malignancies (21), and lung cancer (22) as well as in hospitalized patients (21, 24). The mortality risk associated with GLIM-defined malnutrition has varied from 2.07 in lung cancer (22) to 3.55 in hematologic malignancies (21), risk in line with our results. Lower mortality risk values have been seen among breast, gynecological and colorectal (10), lung (22), and gastric cancer (35) patients, and the mortality risk varies from 1.17 to 1.52 in moderate and from 1.47 to 2.89 in severe malnutrition. Smoking status at the time of HNC diagnosis strongly influences mortality which was seen also in the current study along with malnutrition.

We were not able to show the GLIM criteria to be accurate in predicting survival contrary to two previous studies in patients with variety of cancers (19, 20) and to one study with hospitalized patients (24). Of note, in the Zhang et al. (20) study patients were older than our study population, and in another Zhang et al. (19) study majority of patients (70%) were malnourished, which may partly explain the better accuracy in predicting survival in these studies. Indeed, it has been shown that high age (38) and malnutrition are independent risk factors for mortality (15). Noteworthy, in the study of older cancer patients (20) ROC accuracy was moderate but in another study including a majority of malnourished patients not better than chance (19). In this latter study one probable explanation for the low accuracy is the use of cancer diagnosis as a marker of inflammation instead of CRP, as recommended previously (8, 9, 11). The most likely reason for the low accuracy in our study is the small number of enrolled patients, giving rise to a need for larger multicenter studies. Another explanation might be that malnutrition alone is not strong enough of a predictor for 5-yr survival since other factors like smoking and alcohol abuse are frequently seen among this patient group. Indeed, in the current study heavy smokers had higher mortality risk than malnourished patients. Moreover, GLIM being an objective method compared to subjective PS-SGA method, GLIM may predict better short-term than long-term survival.

We are aware that our research has limitations. First, the GLIM-defined malnutrition diagnostics was performed retrospectively, not at the same time with other nutritional assessments, leading to possible misclassification of nutritional status. Second, given that our findings are based on a limited number of patients, the results from such analyses should be treated with considerable caution. Third, at the time of the original study the PG-SGA translation to Finnish was not performed completely according to the ISPOR Principles which is recommended to perform in future (39). To overcome possible cap in translation process dietitian performed the whole PG-SGA. Strength of the study is that the same research dietitian conducted NRS 2002, PG-SGA, and GLIM-based nutrition diagnostics. In addition, we used objective measures of muscle mass and inflammation to diagnose GLIM-defined malnutrition.

In patients with HNC, the prevalence of malnutrition evaluated by the GLIM criteria is high. These criteria seem to be a potential method for malnutrition diagnostics and outcome prediction in the HNC patient population. NRS 2002 score ≥3 indicates high nutritional risk in this patient group.

## Data availability statement

The raw data supporting the conclusions of this article will be made available by the authors, without undue reservation.

## Ethics statement

The studies involving human participants were reviewed and approved by the Research Ethics Board at Helsinki University Hospital. The patients/participants provided their written informed consent to participate in this study.

## Author contributions

HO was the main investigator, analyzed and interpreted data, and drafted the manuscript. HO, AM, and US designed the study. AM and US supervised the project and assisted with writing the manuscript. PO, AP, and PR assisted in interpretation of the results and writing the manuscript. All authors read and approved the final manuscript.

## Funding

The study was financially supported by HYKS Institute and the Helsinki University Hospital Research Funds.

## Conflict of interest

The authors declare that the research was conducted in the absence of any commercial or financial relationships that could be construed as a potential conflict of interest.

## Publisher's note

All claims expressed in this article are solely those of the authors and do not necessarily represent those of their affiliated organizations, or those of the publisher, the editors and the reviewers. Any product that may be evaluated in this article, or claim that may be made by its manufacturer, is not guaranteed or endorsed by the publisher.
